# Review of the Most Important Methods of Improving the Processing Properties of Starch toward Non-Food Applications

**DOI:** 10.3390/polym13050832

**Published:** 2021-03-09

**Authors:** Arkadiusz Zarski, Krzysztof Bajer, Janusz Kapuśniak

**Affiliations:** 1 Department of Dietetics and Food Studies, Faculty of Science and Technology, Jan Dlugosz University in Czestochowa, Armii Krajowej 13/15 Ave., 42-200 Czestochowa, Poland; arkadiusz.zarski@ajd.czest.pl; 2 Lukasiewicz Research Network—Institute for Engineering of Polymer Materials and Dyes, Marii Sklodowskiej-Curie 55 Str., 87-100 Torun, Poland; krzysztof.bajer@impib.lukasiewicz.gov.pl

**Keywords:** starch, modification, packaging, non-food applications, biodegradable polymers

## Abstract

Starch is the second most abundantly available natural polymer in the world, after cellulose. If we add its biodegradability and non-toxicity to the natural environment, it becomes a raw material very attractive for the food and non-food industries. However, in the latter case, mainly due to the high hydrophilicity of starch, it is necessary to carry out many more or less complex operations and processes. One of the fastest growing industries in the last decade is the processing of biodegradable materials for packaging purposes. This is mainly due to awareness of producers and consumers about the dangers of unlimited production and the use of non-degradable petroleum polymers. Therefore, in the present review, an attempt was made to show the possibilities and limitations of using starch as a packaging material. The most important physicochemical features of this biopolymer are discussed, and special attention is paid to more or less environmentally friendly methods of improving its processing properties.

## 1. Introduction

Humanity increasingly understands that the earth is not an unlimited source of raw materials and that the overexploitation of natural resources causes a lot of damage to the environment. Everyone can agree that the world economy should be increasingly based on renewable raw materials and energy sources. The growing demand for new organic materials and energy demand encourages the search for cheap, widely available biodegradable raw materials and environmentally friendly methods of their processing [1]. It seems more and more certain that the way of dealing with or eliminating the problems of raw materials is through the use of natural polymers based on polysaccharides. Although the vast majority of carbohydrate polymers are used for food purposes, it is predicted that these proportions will change significantly in the coming decades in favor of the synthesis of new functional non-food materials.

The reduction of fossil-fuel resources and environmental problems have contributed to the development of compounds based on natural polymers that can replace traditional petrochemical polymers. Cheap, non-toxic, and biodegradable materials are sought [2]. One of them is starch, the main energy storage in plants and the second-most abundant renewable polysaccharide in the world after cellulose. Starch has long been popular as a raw material for the production of biodegradable packaging. However, the main disadvantage of starch as a potential packaging material is its strong hydrophilicity, high sensitivity to external factors, mainly moisture, brittleness, and very poor miscibility with hydrophobic synthetic polymers. A huge opportunity was seen in the thermoplastic starches (TPS), i.e., in amorphous and homogeneous forms, obtained under the influence of thermomechanical energy during extrusion [3]. This method of plasticizing starch, however, did not solve the problem of brittleness, sensitivity to external factors and poor mechanical properties compared to other polymeric materials. The situation tried to improve with the formation of blends (mixtures) of thermoplastic starch with synthetic, usually aliphatic polymers, for example, polylactide (PLA) or poly-ε-caprolactone (PCL). Still, there was a compatibility problem. The addition of external plasticizers or fillers only slightly increased the processing and usability of starch films [4]. Therefore, to expand the application potential, starch undergoes various types of modifications, from low-complex hydrothermal treatments (like the mentioned plasticization or extrusion) to multistage processes and reactions, using complex technologies [5,6]. Most of them were carried out for hydrophobization. Hydrophobic starch derivatives have usually been synthesized using classic organic/inorganic solvents such as dimethyl sulfoxide (DMSO), dimethylformamide (DMF), *N,N*-dimethylacetamide/lithium chloride (DMAc/LiCl), or pyridine. Mainly their flammability and high toxicity are the features that pose a serious threat during the washing and recovery process and thus can be a considerable and troublesome pollution for the natural environment [7]. For this reason, it has become important to search for a new type of media that is most compatible with the principles of the “green chemistry” as well as the principles of sustainable development. In the last decade, unconventional media (ionic liquids and supercritical CO_2_) and reaction techniques (microwave and ultrasound) have become increasingly popular [8]. Due to the mild reaction conditions and the fact that no harmful byproducts are generated, biocatalysts have also gained great importance in the modification of starch [9,10].

## 2. Structure and Properties of Starch

**Figure 1:**
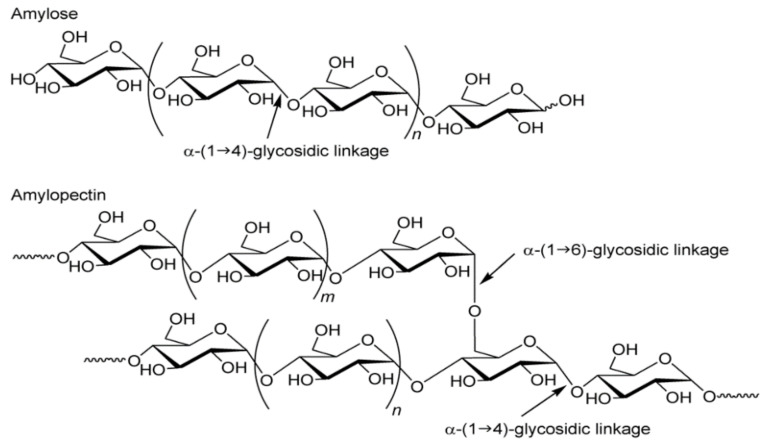
Structure of amylose and amylopectin [15].

Chemically, starch is a homopolysaccharide. It is composed of one type of unit—α-d-glucopyranose, occurring in the form of closed six-carbon rings, cocreating chain and branched forms of the polymer. The α-1,4 and α-1,6 glycosidic bonds dominate in the connections of the polymer glucose units [11]. Such bonds are formed as a result of the joining of neighboring α-D glucose molecules through the oxygen atom cocreating the hydroxyl group, with the release of the water molecule. Glucose polymerization in the starch molecule produces two polysaccharide fractions: linear amylose and branched amylopectin [12]. Both fractions consist of chains composed of α-d-glucopyranose residues connected by α-1,4-glycosidic bonds, while the chains are connected with each other by α-1,6-glycosidic bonds, thus creating branches in polymers (Figure 1). In principle, amylose is considered to be a typical long-chain linear fraction, but few branchings have been proven to occur in it. Both the branched and linear forms of amylose consist of long chains. The second fraction, amylopectin, is characterized by a high degree of branching (approx. 5% of the molecule) and relatively different chains, leading to a highly complex molecular structure. Amylopectin consists of long and short chains. Considering the branching points in addition to the length, we distinguish: the shortest A-chains, without branches, the B-chains that are branched by the A-chains or other B-chains, and the C-chains that contain the only reducing terminal residue and B-chains. The number of short chains as branches are larger than that of long one, but despite this, the size of amylopectin as an integrated structure of many chains is much larger than amylose [13,14].

The occurrence of certain physicochemical features of starch depends, to a large extent, on the genetic conditions, species and varieties of plants from which it was isolated, as well as the conditions of cultivation of these plants. Furthermore, strong differences in physical and chemical properties are dictated by the differences in the content of non-polysaccharide components as well as the structure of starch grains [11].

Native starch is insoluble in cold water and most organic solvents, which is particularly associated with the presence of insoluble or sparingly soluble amylose fraction. As previously mentioned, when discussing non-polysaccharide starch ingredients, one of the above is water. Starchy grains can increase its amount through so-called reversible swelling, manifesting an increase in their volume of up to 30% [14]. Swelling is an exothermic process during which water molecules are absorbed into the amorphous zone, where they are bonded by hydrogen bonds with free hydroxyl groups of the glucose units in polymer chains. However, if the aqueous starch suspension is heated above a certain temperature, the starch granules swell spherically and become amorphous. The above thermal transition is referred to as starch gelatinization, the temperature of which is that of gelatinization [16]. It is a parameter with different values depending on the type of botanical starch. Moreover, using the term “gelatinization” temperature range is more appropriate because the gelatinization of grains depends on their size, from the largest to the smallest, thus gradually proceeding [17]. For example, for potato starch, this range is between 50–70 °C. Gelatinization is accompanied by other phenomena or processes such as loss of birefringence, increase in viscosity, and electrical conductivity. Over time, starch gruel may be subject to retrogradation. This is an irreversible process, manifesting itself in the formation of insoluble amylose aggregates precipitated in the form of dendrites. In other words, it is a type of secondary crystallization by restoring hydrogen bonds between hydroxyl groups accompanied by syneresis (dehydration: molecules get rid of water as a result of reducing and tightening intermolecular spaces).

Another important property of starch, in which it is hard to de facto explicitly include physical or chemical properties, is its ability to form inclusion complexes. The most famous of them is the amylose-iodine complex in the presence of iodide ions, where the internal space of the amylose helix is filled with iodine molecules. The resulting complex ion gives a characteristic dark-blue color, disappearing after the previous denaturation of the amylose chain, occurring, e.g., after heating or hydrolysis. Iodine complexation can be used as one of the faster methods for determining the degree of the depolymerisation of amylose (in shorter chains, the complexes turn red or purple). Amylose can also be complex with other compounds, e.g., organic, to form a characteristic crystallographic structure called “V”. The way of complexing depends on how the structures of the compound fulfilling the role of the “guest” may be different [11].

In turn, the chemical properties of starch, as well as other chemical compounds, are closely related to its reactivity. Starch is a polymer that chemically has most of the characteristics of alcohols, aldehydes, or ethers. The reactivity of native starch is relatively low and mainly relates to the hydroxyl groups of glucose units (substitution and oxidation reactions) as well as glycosidic bonds between individual polysaccharide mers (depolymerization reactions such as dextrinization). It mainly depends on factors such as the type of ring conformation, intermolecular bonds, electron density on oxygen atoms, and spherical reactions with neighboring chemical groups [18]. Among the reactions that starch may undergo, we can generally distinguish two groups, i.e., reactions that cause complete structural disintegration, including the breakdown of glycosidic bonds and granule degradation, and reactions resulting in a small, partial grain distribution, mainly due to the breakdown of intermolecular hydrogen bonds. Technically, due to the complicated granular structure of starch—and thus difficult access to hydroxyl groups and glycosidic bonds—mostly solvent reactions or the initial stage of starch gelatinization are used [19]. Homogeneous reactions, however, are not the only way to the modification of this polysaccharide. In the literature reports of effective solvent-free reactions, these are known to be carried out in a solid phase, heated conventionally or using a microwave radiation [20,21].

## 3. The Methods to Improve Processing Properties of Starch

**Figure 2:**
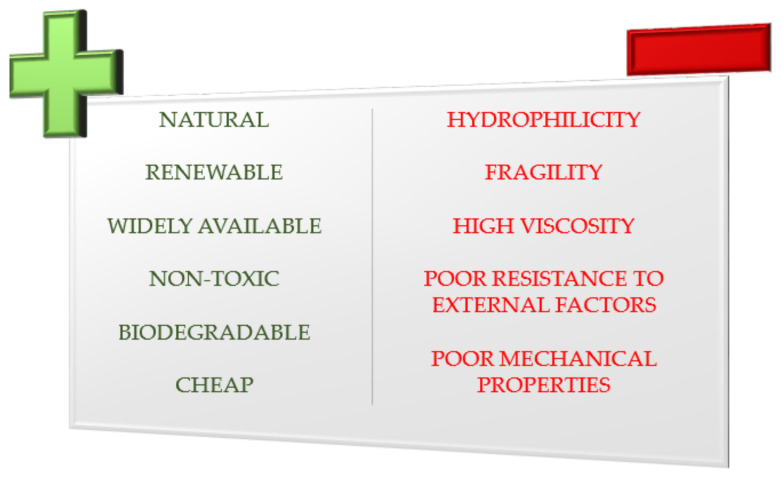
Advantages and disadvantages of starch as a potential packaging material.

Natural starch does not exhibit a good processing property. Its high brittleness, lack of compatibility with hydrophobic polymers due to its highly hydrophilic nature, poor processing quality resulting from its high viscosity, low resistance to external factors during storage mainly moisture are its main disadvantages, constituting a significant limitation in the possibility of use in various industries. However, the unflagging interest in this polymer results from the fact that despite many disadvantages, it has a range of very significant advantages (Figure 2). First of all, it is a natural, renewable, and biodegradable polymer, which is of great importance in the era of searching for environmentally friendly materials and technologies and reducing its pollution. Therefore, native starches undergo various types of modifications, usually to improve mechanical, processing, or utility properties [22]. They can be divided into four basic groups: physical, chemical, biochemical, and genetic modifications, also known as biotechnological. However, additional modification methods are mentioned more often in which more processes and reactions are used in parallel or in series. These are so-called dual and even mixed modifications [7]. The following are methods of starch modification toward non-food applications, mainly relevant to the packaging industry. The focus was on processes and processing increasing its plasticity, compatibility with natural and synthetic hydrophobic polymers, and increasing resistance to external factors, while maintaining the advantages of non-toxicity and biodegradability [22].

### 3.1. Physical Methods

Physical modifications are usually carried out to destroy starch granules (reduce starch granularity) and improve solubility in cold water or other chemical solvents. Most often, such modifications use methods based on the keeping of granular starch under various conditions of humidity, pressure, temperature, irradiation, or various mechanical systems. Physical methods cause structural changes in starch through the use of mechanical, magnetic, electrical energy, or the combinations thereof. Structural changes result in changing the properties of the entire polymer [23]. During physical modifications, the integrity of the molecules can be preserved (hydrothermal processes), interrupted, or destroyed (loss of granularity with partial depolymerization of the components). The most popular hydrothermal processes include pre-gelation, leading to increased water absorption capacity or “cold” solubility, which can be carried out using the following techniques: spray drying, drum drying, or extrusion [24].

**Figure 3:**
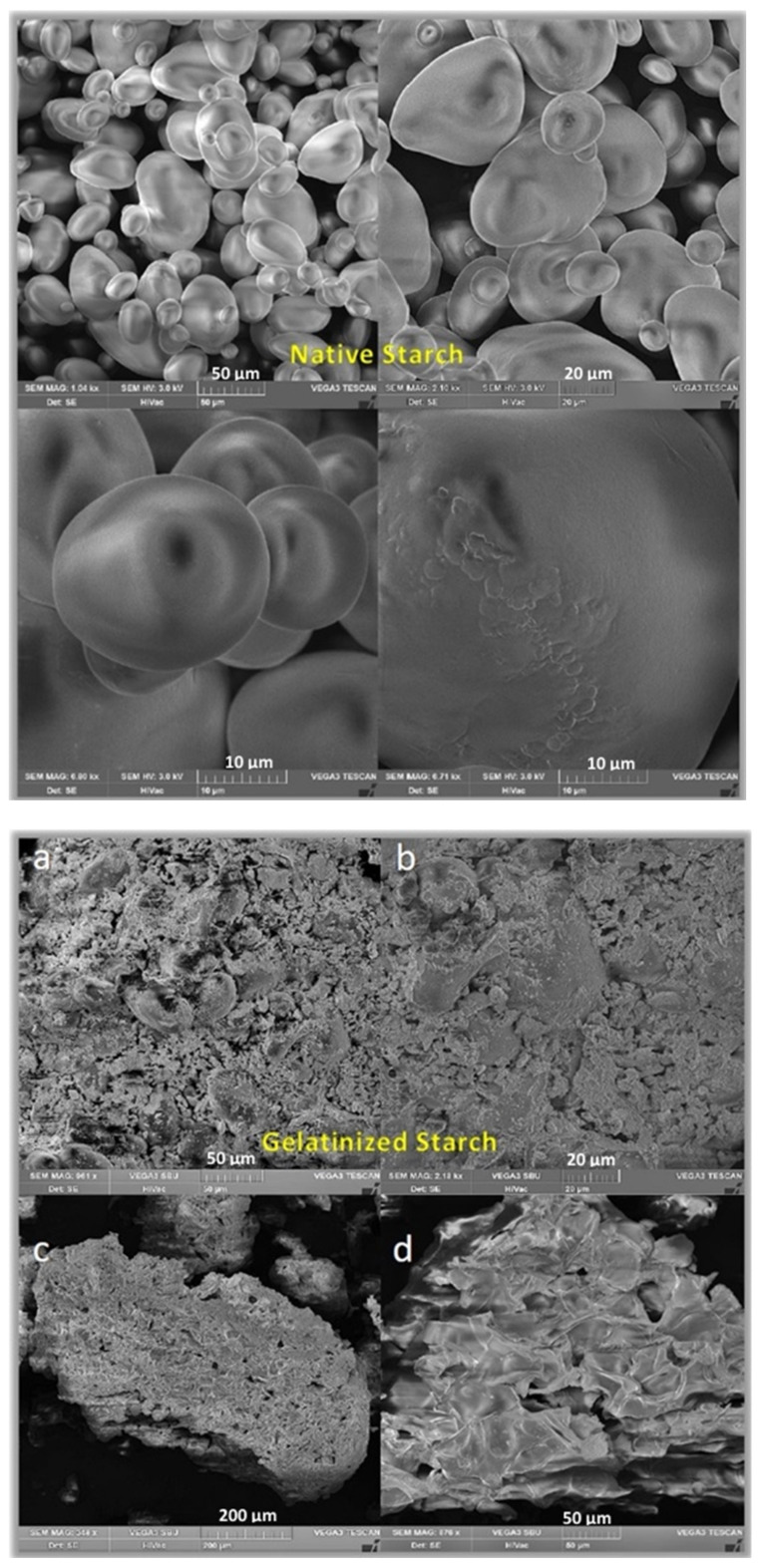
Starch before aand after gelatinization (**a**,**b**)—partial; (**c**,**d**)—full; images made by authors of manuscript, Jan Dlugosz University, Czestochowa, 2020).

The gelatinization process described earlier is also a process classified as the physical modification of starch, which, by rearranging intramolecular and intermolecular hydrogen bonds between starch and water, leads to changes in both the organization of starch grains and changes in many other properties [25,26]. Loss of granularity is confirmed by the swelling of the grains, increase of their volume and loss of crystallinity and hence birefringence (Figure 3).

Hydrothermal starch modification is carried out mainly for the purpose of reducing the possibility of leaching amylose, reducing the viscosity or swelling of grains, and increasing the gelatinization temperature, thermal stability as well as susceptibility to acid and enzymatic hydrolysis, e.g., with α-amylase [27,28]. A characteristic feature of hydrothermal treatments is the change in the physicochemical properties of normal starches without destroying its granular structure, as well as the fact that they occur at temperatures below the pasting temperature and above the glass transition temperature. Both are included in this type of modification—hydrothermal treatment and annealing, carried out at specific humidity levels and appropriate temperatures and times. The first applies to starch modification at low moisture levels, i.e., <35% *w*/*w*, while the second applies to the average values of 40–45% *w*/*w* or high <65% *w*/*w*. Annealing is a method of conditioning starch granules that does not lead to changes in their morphology but indirectly affects the properties of starch such as the ability to gelatinize, swell, or leach depending on the applied process conditions and the botanical source of starch. The main purpose of annealing is to approach the glass transition temperature, which increases the mobility of polymer particles [29,30].

Among other physical methods of starch modification that are increasingly used today, the following can be mentioned: iterated syneresis [31], repeated freezing and thawing [32], processing in a pulsating electric field [33], osmotic pressure treatment [34], or corona electric discharge [35]. Examples of the use of mechanical interactions, high hydrostatic pressure, ultrasound, or electromagnetic rays have been described in the literature [36]. In high-pressure methods, pressures in the range of 400–900 MPa at room temperature are used. Such high pressure limits the swelling ability and thus reduces the viscosity of the starch gels. In addition, in pressure technologies, gases in ionized form are used, i.e., plasma. Ionization is mainly subjected to argon, hydrogen, oxygen, ethylene, or methane. The most frequently observed effects of pressure treatment include changes in the degree of polymerization, hygroscopicity, and oxidation of starch [37,38]. In order to make starch hydrophobic by physical methods, Bastos et al. processed corn starch with plasma and sulfur hexafluoride (SF_6_). The hydrophobization efficiency of the polysaccharide surface, measured dynamically by assessing the contact angle against water, was strictly dependent on the power of the plasma used. Consequently, longer exposure times led to almost complete disappearance of the interface between phases, and surface modifications led to rough short-range corrugations [39].

During microwave exposure to starch, the most important parameters are temperature and humidity, which determine the dielectric properties of starch. Molecular rearrangement occurring during irradiation leads to changes in rheological properties, gelatinization temperature, enthalpy, swelling capacity, and solubility, while causing significant changes in the crystallinity and morphology of starch granules [40]. Ultrasonic treatment of starch, in turn, leads to changes in its amorphous areas while maintaining the shape and size of the granules [41]. Therefore, pre-gelatinized starch is usually subjected to ultrasound methods, where after modification, the surface becomes porous and deformed, and starch properties such as solubility, swelling, and viscosity were changed [42,43].

The most commonly used treatments are based on mainly mechanical interactions include milling, mixing (blending), and extrusion. Ball milling is based on friction between the grains between each other, the mill wall and the balls, which causes them to break up into smaller fragments; in other words, it is their mechanically induced destruction. Damage to the grains during milling results, apart from fragmentation, in lowering the enthalpy of gelatinization, glass transition temperature, and apparent viscosity. Moreover, it induces partial gelatinization of grains by increasing their solubility in cold water and its absorption [44,45].

**Figure 4:**
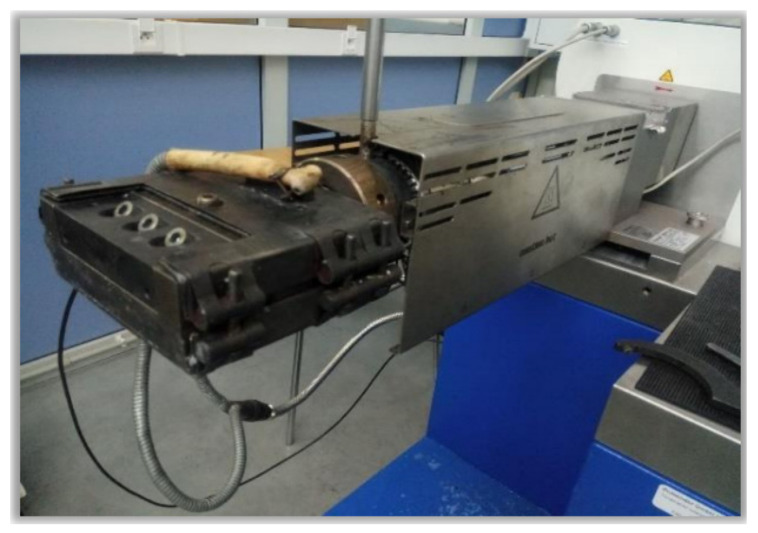
The single-screw extruder (Brabender^®^, Duisburg, Germany), equipped with a narrow slot head (photo taken by authors of manuscript, Lukasiewicz Research Network, Poland, 2018).

The starch decomposition temperature is lower than its melting point (about 220 and 240 °C, respectively). Therefore, to reduce the latter, compounds with plasticizing properties are used, such as polyols (glycerol, sorbitol, and polyvinyl oxide), mixtures thereof, as well as compounds containing nitrogen, urea, biuret, amine derivatives, and ammonium derivatives [46]. Furthermore, plasticization is a common procedure preceding extrusion or high-pressure injection. Extrusion is one of the most commonly used methods in material processing to obtain thermoplastic products, both based on synthetic, semi-synthetic, or even natural polymers. In the case of a polymer, which is starch, it is a process where, acting on it with thermomechanical energy, it breaks down its granular and semicrystalline structure, resulting in an amorphous and homogeneous material known as TPS [47]. Most often, extrusion is carried out in one or two stages in devices called extruders, with various more or less complex structures. In a one-stage process, the twin-screw extruder is usually filled with native starch, and the plasticizer is added in portions during extrusion. When the extrusion takes place in two stages, the dry mix is first prepared, i.e., the extrusion is preceded by plasticization in a mixer to bring about homogeneous polydispersity. Generally speaking, extrusion consists of plasticizing the material and then extruding it through channels of a given profile. Extruders are composed of three basic systems: drive, control, and plasticizing, and the latter of several zones, such as the supply, compression, and dosing zones and several heating zones along the cylinder (Figure 4) [48]. The material is heated to achieve a certain plasticity and in the next stage, it is conveyed using a screw conveyor (the so-called screw) through subsequent zones up to the extrusion head and then cooled, obtaining a presswork (e.g., moldings, films). Heating, plasticizer, or finally shear forces (internal friction forces in the cylinder) used during extrusion may not only lead to the destruction of starch grains and their fragmentation but also to its uncontrolled partial depolymerization [4].

The improvement of physicochemical properties of starch can be obtained by blending it with other natural polymers or, more often, synthetic polymers. Starch in granular form has already been used several times as a type of filler that makes it possible to increase the biodegradability of widely used plastics, including linear low-density polyethylene (LLDPE), high-density polyethylene (HDPE), polypropylene (PP), or polystyrene (PS) [6]. In order to increase the compatibility of starch with hydrophobic polymers, they are often subjected to physical, chemical, enzymatic, or mixed initial modifications.

Usually, mixtures with a content of starch or its derivatives from 6 to 20% are prepared, however, mainly to improve the properties of the component with a higher content, in this case, synthetic polymers. The addition of polysaccharide promotes better water-vapor permeability, increased dimensional stability during injection molding, and increased stiffness of materials during blow molding [6,49]. For packaging, in the production of mulch films and geotextiles, starch blends with copolymers of ethylene and acrylic esters as well as methacrylates or vinyl acetates are used. Moreover, polar copolymers in mixtures with starch play the role of homogenizing agents due to the reduction of interfacial energy between polysaccharide and polyolefins. In order to use starch with more than 20% in the mixture, it must be subjected to a thermal or chemical pretreatment. Due to which, the starch loses its granular structure and, as a result, forms a continuous phase with the rest of the components in the extruded matrix, for example, poly (ethylene-co-acrylic acid) (PEAA) blend and starch with up to 50% content, using urea as a plasticizer [50].

Starches used as a filler in traditional plastics, especially polyolefins, help to reduce production costs and increase its biodegradability and rigidity. It should be noted, however, that the content of starch in blends is limited to a certain range above which it drastically deteriorates, primarily in performance. Complete disintegration of this type of blend can be achieved using transition metal compounds soluble in the thermoplastic matrix as pro-oxidative additives, catalyzing photo- and thermo-oxidative transformations [51,52,53,54]. It is worth emphasizing here that blending starch with polyethylene or other synthetic polymers over the decades has turned out to be a completely misplaced idea. Hybrid materials based on biodegradable natural polymers and non-degradable synthetic polymers are one of the worst solutions for plastics processing, mainly due to the generation of microplastics. The abovementioned disintegration of such systems through the use of photo- and thermo-oxidative transformations turned out to be a pseudoecological approach. Such materials do not fully degrade but rather disintegrate, generating these microplastics. If we compare them with non-integrated material, they spread more easily in the environment and cause greater damage, especially among living organisms. Therefore, they do not meet any standards related to environmental safety which are increasingly established under international regulations. For example, one of them is the “single-use plastics directive” (SUPD) which puts in place more responsibility for plastic producers and new recycling targets, in this case for EU Member States [55].

Through the years, not only water [56,57], glycerol [58,59,60,61], and sorbitol [62] have been used as plasticizers in mixtures with polymers but also other compounds such as citric acid [63], formamide [57], and urea [64,65]. According to some sources, TPS in combination with other polymers forms one of three types of materials in which: (1) it is incompatible with synthetic polymers (aliphatic polyesters); (2) partly compatible and complexed with incompatible or partly compatible synthetic polymers; and (3) complexed with synthetic hydrophilic–hydrophobic copolymers [3].

The properties of starch composites with other polymers can be improved (greater compostability, better mechanical properties, and weaker sensitivity to external factors, mainly humidity) through the use of a compatibilizer, the presence of polymer chain extenders (epoxides and diisocyanates), or polyesters grafting. Furthermore, compatibilizers increase the adhesion between the polymer phases forming the blend, stabilize the dispersed polymer phase, and protect against proliferation during relaxation. In recent years, many attempts have been made to blend starch with polymers and copolymers of various structure and properties, both with and without compatibilizers. TPS blends with different density polyethylene (LDPE and HDPE) which have been obtained as being more or less compatible [53,66,67,68,69,70,71], TPS blends with PP [60,72], with PS [73,74], with an ABS terpolymer, i.e., acrylonitrile butadiene styrene copolymer [53], with polyvinyl alcohol (PVA) [75,76,77,78], PLA [57,79,80], PCL [81,82,83,84], with natural gum blends [85], with polyamide (PA) [86], with poly(hydroxyl ester ether) (PHEE) [87], poly(butylene succinate) (PBS) [53,88], polyesteramides (PEAs) [89] and polyurethane (PU) [90]. More attention has been paid to this issue in the discussion of starch-based materials for the packaging industry.

### 3.2. Chemical and Biochemical Methods

Chemical modifications are based on introducing a group or functional groups into starch molecules, resulting in clear changes in physicochemical properties and significant differences in application possibilities (Table 1). Chemical methods are preceded or combined with physical methods, such as, irradiation, ultrasound or microwave treatment, as well as mechanochemical methods as is the case with the so-called reactive extrusion [90]. In chemical modifications, depending on the chemical properties of the reagents used, we can distinguish single-function and bifunctional reagents. Monofunctional reagents introduce into the polymer molecule a hydrophobic, non-ionic, cationic, or covalently reactive substituent group, and more than one of such groups in bifunctional. Monofunctional groups are used, e.g., during esterification or etherification, while bifunctional ones, reacting due to their nature with more than one hydroxyl group enable, e.g., the crosslinking of polymers [5,91,92].

Starch can be crosslinked with various reagents, including epichlorohydrin, phosphorus oxychloride, sodium trimethyl phosphate, and sodium tripolyphosphate. During crosslinking, bonds occur between starch hydroxyl groups and a group or functional groups of crosslinkers, as a result of which, a characteristic dimensional network structure is created [93,94]. The crosslinking of starch improves its processing properties and stability in an acidic environment [95].

In turn, graft copolymers are constructed in such a way that lateral branches are constructed of other units than those which form the main chain are attached to the main starch polymer chain. Different research groups have tried to introduce synthetic or natural polymers into the skeleton with more or less success. However, evidence has so far shown that this copolymerization usually begins at the end groups at C-1 or C-2 carbon [96,97]. Among the described and used mechanisms of this type of modification, we can distinguish ionic condensation or an even more popular free radical mechanism. Cerium salts such as cerium ammonium nitrate, potassium permanganate, potassium persulphate, benzyl alcohol, and benzoyl peroxide have proved to be effective copolymerization initiators, and as monomers: acrylamide, acrylonitrile, methacrylamide, vinyl alcohol, L-lactide, acrylic acid, and p–dioxanone [5].

Oxidation of starch is carried out by treating it under various conditions with various oxidants such as potassium permanganate, iodates (VII), sodium chlorate (I), hydrogen peroxide, or persulphates. During oxidation, starch undergoes significant depolymerization due to the progressive hydrolysis of glycosidic bonds. Hydroxyl groups (mostly groups at C-2, C-3, C-6 carbons) are first oxidized to carbonyl groups and then to carboxyl groups [3,98]. Furthermore, dialdehyde starches have been obtained using a very strong oxidant—iodic acid (VII), with the potential for direct oxidation of adjacent hydroxyl groups. Oxidation makes it possible to increase thermal stability and improve the mechanical properties of starch, while reducing its ability to absorb water, which is very desirable for the use of this type of material in various industries, especially packaging [98]. In recent years, many research centers around the world have attempted to replace chemical starch oxidants with ozone as a “green” alternative to reduce the generation of harmful byproducts [5,99,100].

Chemical modifications in which the hydroxyl groups of starch are replaced by a carboxymethyl, hydroxypropyl or hydroxyethyl group through the formation of an ether bond are called “etherification”. Many etherification procedures are known, but most combine the use of an alkaline, basic catalyst, usually NaOH, to initiate the reaction [101,102]. Carboxymethylation, due to the speed and simplicity of the reaction, is one of the most commonly used methods of etherification, during which the hydroxyl groups of starch are replaced by anionic carboxymethyl groups. In addition, carboxymethyl starch is characterized by increased hydrophilicity and water absorption capacity [103,104]. Alkaline-catalyzed etherification of starch with propylene oxide leads analogously to obtaining hydroxypropyl starch derivatives in nucleophilic substitution reactions of polymeric hydroxyl groups. Compared with carboxymethyl, hydroxyl starch derivatives have a more disintegrated semicrystalline structure, absorbing higher amounts of H_2_O [102,105]. Highly water-soluble hydroxyethyl derivatives of starch are obtained in a similar manner as hydroxypropyl, with the difference being that the etherifying agent is ethylene oxide [106].

A large number of hydroxyl groups in the starch polymer chain give it the character of a polyalcohol, which may also undergo another substitution reaction, namely esterification. For each anhydroglucose unit, there are as many as three OH groups (at C-2, C-3, and C-6 carbon) with the possibility of substitution. There are basically two types of starch esters—inorganic and organic—depending on the esterifying agent used [5]. Esterification is one of the oldest methods of starch hydrophobization, which gives enormous application potential to this polymer. Esterified starches have been obtained for decades mainly using traditional technologies like convection heating, in solid phase, or in classic organic solvents. It is a modification primarily based on chemical methods, although more and more often, dual and even mixed methods are promoted in publication reports and patents. The more or less successful attempts of starch ester synthesis preceded by physical hydrothermal modifications, using enzymatic catalysis, as well as innovative methods and techniques, new type of reaction media, such as ionic liquids, supercritical CO_2_, synthesis in the conditions of a microwave radiation, using ultrasound or mechanochemistry were carried out [7,107]. The efficiency of starch modification through esterification is influenced by several factors such as its botanical variety, concentration of reagents, reaction time and temperature, pH of the reaction system, the presence of catalysts, activators, and inhibitors [8]. Appropriate control of the esterification conditions allows the synthesis of starch esters with different degrees of substitution, thus different mechanical, processing and functional properties, widely used in both the food and non-food industries.

Esterification can occur directly when the agent is inorganic or organic acid, or indirectly by reaction with acid anhydrides or acid derivatives. Esters of starch and inorganic acids such as sulfates, nitrates, phosphates, and borates are well known from the available literature. By heating starch with urea (or other compounds such as formamide, dicyandiamide) in the reactions of starch with a mixture of phosphoric acid, magnesium sulfate and amides, carbamates, and starch phosphates with strong binding properties are obtained, used in the paper industry and as an adhesive [108]. Starch nitrates can be obtained using a nitrating mixture or nitrogen oxide (V) vapor in an air stream. Starch phosphates, synthesized by covalent bonding of orthophosphorus residues can be divided into mono and distarch. Monostarch phosphates are formed using hydrogen phosphates and dihydrogen phosphates (V) as reactants at 120–170 °C, and distarch using a crosslinking agent, such as POCl_3_ (phosphoryl trichloride), where orthophosphorus moieties act as a link between adjacent polymer chains. In turn, starch sulfates are synthesized by reactions with sulfur, sulfur oxide (VI) or sulfuric acid (VI), as well as chlorosulfonic acid and its derivatives [5,108]. In addition, starch xanthates were obtained in reactions with carbon disulphide in alkaline medium, and xanthid starch was obtained in subsequent stages by oxidation with hydrogen peroxide or sodium chlorate. Inorganic esters of potato starch have also been synthesized by reactions not only with the appropriate acids, but anhydrides, salts, and hydrosols of these acids, among which sulfates, silicates, boric acid esters, selenates, zincates, starch cuprates, and silicates are present [21,109,110,111,112,113,114,115]. Hydrophobic starch derivatives have also been obtained by benzylation and benzyl chloride using one of two methods, i.e., surface benzylation of starch grains in heterogeneous modification or homogeneous modification in an alkaline environment [116].

One of the first ways to synthesize organic starch esters was to react with short-chain carboxylic acids (C-1 to C-6). Initially, esterification was carried out not only with fatty acids such as butyric, caproic, and valerian, but also with anhydrides such as acetic or octenyl succinic acid; the obtained products showed the properties of thermoplastic materials [8,117]. The synthesis of long-chain starch esters was proposed by Aburto et al. and Thiebaud et al. using appropriate acid chlorides and pyridine as the reaction medium [118,119]. They proved that with the increase of the acyl chain length and the degree of substitution, the hydrophobicity of the obtained compounds increased, while their water absorption and biodegradability decreased. With time, the use of pyridine and chlorides began to be abandoned in favor of not only higher carboxylic acids, but also their esters [8]. Starch transesterification reactions with stearate or vinyl laurate are known in the DMSO environment as a solvent and basic salts as catalysts (sodium acetate and hydrogen phosphate, potassium carbonate) [120]. DMSO also proved to be a good reaction medium for the esterification of starch with fatty acids catalyzed by potassium persulphate [121] and using other esterifying agents such as acid chlorides, methyl esters, or acyl imidazoles [122,123]. The role of solvents during esterification, as well as other starch modifications, consists of loosening the polymer structure (breaking hydrogen bonds), thus increasing the availability of reagents to free OH groups. However, the classic organic solvents used so far such as DMSO, DMF, pyridine, or tert-butanol, have some disadvantages and limitations, namely toxicity and flammability, thus negatively impacting the environment. Therefore, over time, the search for new media and reaction techniques, more environmentally friendly and in line with the principles of sustainable development, have begun. Examples of such media can be supercritical CO_2_, ionic liquids, techniques of biocatalysis, and reactions carried out under microwave irradiation, in the presence of ultrasound or mechanochemistry [7]. Moreover, starch esterification was carried out using the method of dialysis or reactive extrusion [124,125]. Solvent-free synthesis of starch esters was performed with the use of octane, octadecane, decane, dodecane and tetra, and hexadecanoate chlorides in the presence of formic acid as a factor initiating hydrogen bond breaking and esterification [8,119]. In the following sections, the most important methods of starch modification were reviewed (Table 2, Table 3, Table 4 and Table 5) and then their main advantages and disadvantages were compared (Table 6).

In addition, attempts have been made to conduct esterification of starch in the presence of biocatalysts, both in methods with or without solvent, with conventional or microwave heating. The esterification of starch using enzymes as catalysts is an environmentally friendly method due to the mild conditions of synthesis, non-generation of onerous byproducts, and the possibility of conducting stereo and regiospecific reactions [126]. The use of lipases as catalysts for the esterification of starch with fatty acids is one of the newer alternatives to its modification through hydrophobization. Lipases are a very interesting group of enzymes due to their unique catalytic properties. In the aqueous environment, these enzymes catalyze the hydrolysis and breakdown of triacylglycerols to diacylglycerols and monoacylglycerols or fatty acids and glycerol. It is a reversible reaction, implying that under anhydrous conditions, the enzymes can catalyze the reverse reaction, i.e., the synthesis of esters. Commercially available lipases are isolated from bacteria, for example, *Staphylococcus aureus*, *Burkholderia,* or the genus *Pseudomonas*, although more often from fungi such as *Candida* or *Thermomyces* [7,8].

Enzymatically catalyzed esterification of starch in a water bath was carried out in solvent-free system or in an environment of organic solvents such as DMSO, DMF, and tert-butanol or acetone, as well as in an alkaline environment or in the presence of a surfactant [8,107]. The esterification carried out under the conditions of microwave electromagnetic radiation enables the synthesis of hydrophobic derivatives of starch under much more economical conditions, i.e., shorter time and lower temperatures compared to methods based on conventional heating. Microwave heating may not be an optimal method of energy transfer to the system, but it allows efficient heating from the inside, owing to the coupling of microwave energy with the components of the reaction mixture. For the first time, such reactions were carried out to synthesize tapioca starch succinates or starch maleate [127,128]. Other examples include the esterification of maize starch with acetic acid and anhydride, heterogeneous esterification of tapioca starch with citric acid [129], or transesterification of bean starch with vinyl acetate, catalyzed with potassium carbonate [8]. Starch acetates were most commonly obtained using microwaves, where the esterifying agent was an acid anhydride with the addition of acetic acid as a solvent, and the reactions were catalyzed by molecular iodine. Highly substituted starch derivatives were obtained by Diop et al. during iodine-catalyzed esterification of corn starch with a mixture of anhydride and acetic acid using microwave assistance [130,131]. A wide range of esters, with different degree of substitution depending on the reaction parameters, were also obtained in an aqueous environment using chemical catalysts such as K_2_CO_3_, NaOH, or a biocatalyst such as bacterial lipase from *Staphylococcus aureus* (Table 2). Another successful attempt was also made to perform other biocatalysts of microwave-induced electromagnetic induction, namely corn and tapioca starch. In said reaction, hydrolyzed oils and free fatty acids were used as acyl donors and enzymes of the type of lipases of fungal or bacterial origin as catalysts, resulting in products with different properties depending on degree of substitution [8]. For comparison purposes, potato starch was esterified with oleic acid without solvent under microwave heating and additionally using an organic solvent DMSO, in a water bath. In both cases, the catalyst was fungal lipase from *Candida antarctica* in immobilized form on a polymer carrier [132].

An interesting idea was to carry out esterification or etherification in ionic liquids (ILs). These are compounds with ionic structures, more specifically consisting of an organic cation and an organic or inorganic anion. Despite this structure, they are not typical salts, because their melting point is lower than 100 °C. Their unusual properties and considerable design possibilities through structural modifications give them a considerable advantage in competing with classic organic solvents. They are non-volatile compounds with high durability and thermal inertness, characterized by high polarity, electrical conductivity, and a wide range of electrochemical stability. Multifunctionality is also a feature of ILs that distinguishes them from other solvents [138,139,140]. Ionic liquids can dissolve, among others inorganic, organic, organometallic compounds (catalysts), and polymers [141,142]. Most reports on the possibility of carrying out modifications of carbohydrate polymers (mainly cellulose and lignin) using them as a reaction medium and solvent have appeared over the last 20 years. One of the first applications of this type of compound for starch was the synthesis of its acetates in 1-butyl-3-methylimidazolium chloride, additionally catalyzed with pyridine. To date, the chloride is the most commonly used liquid in the esterification and transesterification reactions of starch of various origin, i.e., using anhydrides, acids, esters, non-catalyzed, or chemically or enzymatically catalyzed. In addition, 1-allyl-3-methylimidazolium chloride ([AMIM]Cl), 1-butyl-3-methylimidazolium chloride ([BMIM]Cl), acetate ([BMIM]OAc), hexafluorophosphates ([BMIM]PF_6_), tetrafluoroborates ([BMIM]BF_4_), and others have proved to be effective starch solvents. Ionic liquids, as well as other solvents, are used in starch modifications in order to loosen its macrostructure by breaking inter- and intra-molecular bonds, thereby increasing its reactivity [143]. In order to shorten the time of partial or total starch placement, reactions in ILs can be carried out, as in other modifications, in a bath or a microwave reactor. The literature reports from the last few years show that ionic liquids can be used for efficient biocatalysis [7,8]. In addition, it is worth noting that although they proved to be sufficiently good solvents and reaction media for starches of various botanical origins, for the purposes of esterification and transesterification, they were used mostly for corn starches, with different content of individual fractions, in reactions without the use of a catalyst or catalyzed by pyridine or lipases (Table 3) [144,145,146].

Supercrtical CO_2_ (scCO_2_) is a non-polar, non-toxic, and chemically inert solvent and easy to remove from the reaction system by expansion. In particular, CO_2_ in the supercritical state is capable of lowering the starch gelatinization temperature through diffusion into its interior and deformation of crystalline areas. The scCO_2_ as the solvent was used for the first time in the transesterification of potato starch with vinyl or methyl esters of fatty acids in the presence of basic salts, as well as esterification with anhydrides (Table 4) [162,163,164,165].

Ultrasound was mainly used during the esterification of starch with monochloroacetic acid in a mixture of ethanol-water and NaOH-catalyzed solvents, and in the synthesis of acetate and octenyl succinate of starch catalyzed with H_2_SO_4_ and K_2_CO_3_, respectively [167,168]. Octenyl succinates of waxy rice starch and corn starch were obtained in chemically catalyzed and ball-milled esterification reactions in both aqueous and anhydrous environments. Mechanochemical methods have so far been used successfully mainly in esterification of cassava and waxy rice starch (Table 5).

Hydrophobic derivatives of corn starch were also synthesized by esterification with dodecenyl succinic anhydride (DDSA). Chi et al. conducted an alkaline catalyzed reaction of pre-emulsified DDSA anhydride with granular starch [173]. In another research group, the hydrophobicity of corn-starch film was increased by modification with not only dodecyl succinic anhydride but also octenyl succinic anhydride [174]. Hydrophobic starches in foamed form have also been successfully obtained, which was done by Tahlawy et al. by emulsifying cooked corn starch and alkyl ketene dimer under high shear [175]. However, the hydrophobic character given to the foams was not sufficient to slowly wet the porous surfaces of such materials. In 2007, Barikani and Mohammadi received new polyurethane starch derivatives with increased resistance to external conditions, especially moisture [176]. They carried out two-stage reactions with prepolymer, consisting of isocyanate-terminated PCL. The hydrophilicity of coatings based on thermoplastic starch was also reduced by Carvalho et al. through surface treatment with phenyl isocyanate (PhNCO), polyisocyanate blocked with phenol trimethylolpropane and toluene diisocyanate (TMP-TDI-phenol), copolymer of styrene and glyceryl methacrylate. and stearoyl chloride (StCl) [177]. Limited surface modification has allowed authors to improve performance while maintaining material biodegradability.

Starch esters and other hydrophobic starch derivatives synthesized in various ways were primarily used in the production of various types of mixtures with synthetic polymers. For example, the blends obtained by the process of reactive extrusion of acetylated and oxidized banana starch with LDPE. Owing to the use of reactive extrusion, mixtures were also made of thermoplastic corn-starch maleate with poly (butylene adipate-co-terephthalate) (PBAT) and acetylated cassava starch with an aliphatic polyester–polyurethane [178]. The production of mixtures preceded by chemical or biochemical modifications of starch is not only intended to increase compatibility with other polymer components but primarily to increase the hydrophobicity, thermoplasticity, and mechanical resistance of the resulting blends.

## 4. Starch-Based Materials for the Packaging Industry

**Figure 5:**
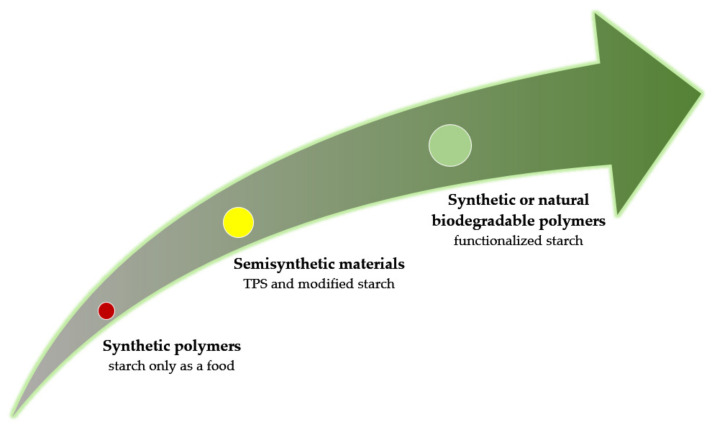
Evolution of packaging materials and starch application.

For decades, plastics have been considered a boon to the modern world. Only recently, among the society as a consumer, awareness arose about the threat and problem of their high resistance to degradation. The environmental impact of sustainable plastic waste is a matter of general concern around the world, and disposal methods for waste are still insufficient. Space for landfills are limited, combustion utilization can generate toxic air pollutants, and mixed-waste recycling methods are often energy consuming and therefore not cost effective [47]. In addition, it is worth realizing that the majority of synthetic polymers are petroleum compounds, and as it is well known, oil reserves are decreasing year by year. Reusing and recycling petroleum polymers through processes such as milling and secondary extrusion may be cost effective, but they are not a very good solution due to loss of functional properties or deterioration of mechanical properties of strategic importance. In the face of the above problems, it will be important to find durable substitutes for plastics, especially for the production of disposable materials and short-term packaging. That is why the interest in exploring the possibility of using biodegradable and renewable polymers instead of widespread non-degradable polymers such as polyethylene, polystyrene, etc., has become prevalent. Currently, the approach in the packaging industry is to make fashionable the use not only native or modified but functionalized ecofriendly polymers. Another trend is to focus on designing multilayer biodegradable packaging with improved barrier properties against both biotic and abiotic external factors [179]. In this way, starch, usually associated with rather food applications, also attracts attention (Figure 5).

### 4.1. Starch and TPS as a Packaging Material

Among biodegradable polymer materials for packaging, starch is the most popular, just after cellulose [180]. This polymer has a sufficiently good barrier against gases such as O_2_ and CO_2_. The direct use of native starch or even thermoplastic starch as raw materials for packaging production is limited by their fragility, lack of compatibility with hydrophobic polymers, high moisture sensitivity, and poor water-vapor barrier, as well as low resistance to mechanical factors such as tearing and picking [181]. Starch coatings are highly hydrophilic, and products made of it quickly swell and undergo significant deformation in conditions of high humidity. Therefore, to improve its properties, research has been conducted for many years not only on structural modifications of starch but also on the development of physical mixtures with hydrophobic synthetic polymers, to lead to a simultaneous reduction in their consumption as well as an increase in biodegradability. Thus, starch-based packaging may contain its native, thermoplastic, or modified form. Treatments aimed at improving its processing and utility properties, and thus increasing competitiveness with widely used synthetic polymers are usually based on mixing (blending) and synthesis of its derivatives (derivatization). Packaging based on natural polymers is obtained by the same techniques as for synthetic polymers, i.e., by casting, extrusion, or blowing. In the 1980s of the last century, attempts were made to create blends of starch and synthetic polymers in the extrusion process, and in the next decade the first procedures for obtaining packaging materials based on pure modified starches were proposed [47].

**Figure 6:**
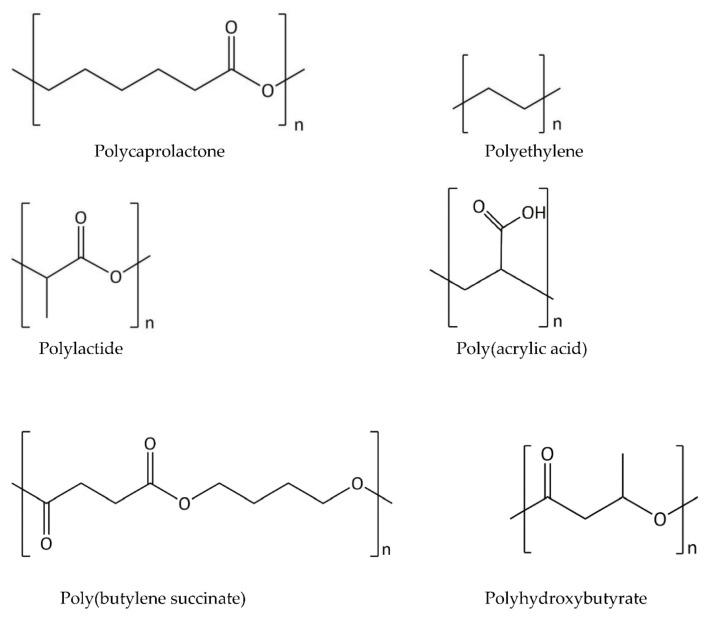

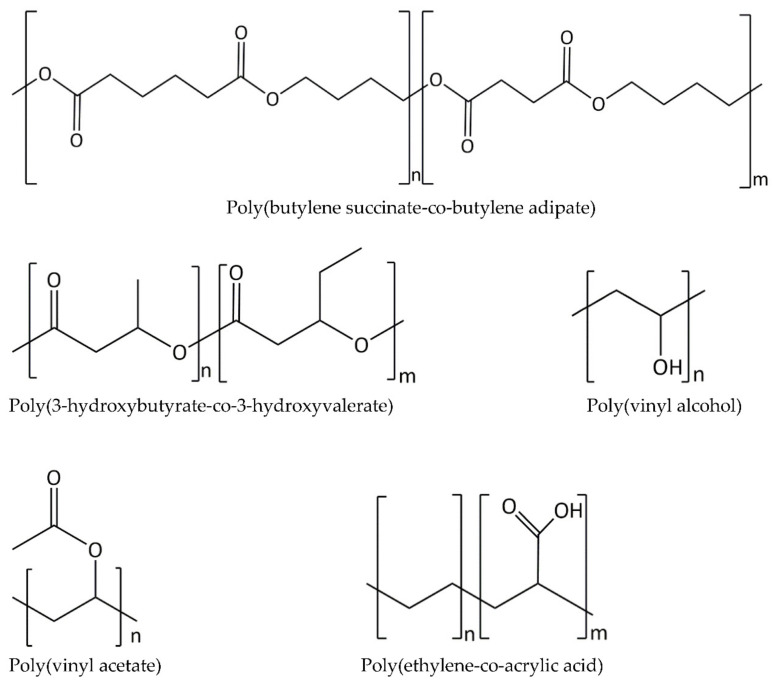
The name and structure of the most common polymers used in blending with starch or thermoplastic starch (TPS).

Starch-based packaging is made in most cases from corn and potato starch, which may not be surprising due to their significant resources around the world. The use of high amylose corn starch as a packaging material compared to normal starch enabled the development and obtaining of films with higher barrier and mechanical strength. One of the oldest and generally the most commonly used methods to date of using starch as a packaging material is blending it with other natural or synthetic polymers (Figure 6, Table 7). Mixtures (blends) obtained in this way constitute a material with aggregate benefits, which are not only biodegradable, but moreover with improved mechanical properties and, most importantly, much cheaper. The combination of starch and synthetic polymers in the form of blends is carried out to overcome its limitations in use, resulting from its fragility, sensitivity to moisture, and poor mechanical properties. The first attempts at mixing normal starches and polymers, due to the hydrophilicity of the polysaccharide and thus poor miscibility, did not yield the expected results: after mixing, the products were delaminated and showed poor interfacial properties. The blending of starches with moisture-resistant hydrophobic polymers made it possible to obtain a biodegradable polymer mixture with sufficiently good mechanical properties. The situation also improved when high amylose starch and gelatinized starches were used [182]. In subsequent trials, the use of plasticized starch was chosen. As with the processing of other polymers, TPS is obtained mainly using extruders and injection molding machines [183]. Plasticizers at high temperatures (90–180 °C) transform granular starch into a homogeneous and plastic form, thus enabling its extrusion, pressing or injection molding, as well as dissolving and lowering the melting point. The breakdown of starch granules contributes to increasing the mobility of the macromolecular chain, which results in the material softening and becoming less brittle. In this way, semicrystalline, highly organized starch grains, are transformed into an amorphous and homogeneous thermoplastic material. Plasticizers can be divided externally, somehow added from the outside and competing with hydroxyl groups of starch for coformation of hydrogen bonds, and internally when the derivatization of starch is based on the substitution of its hydroxyl group. It thus induces the destruction of existing hydrogen bonds. Various mechanisms and theories of plasticization have been proposed, e.g., lubrication theory, gel theory, or free volume. The theory of lubrication assumes that the plasticizer accelerates the mobility of starch molecules among themselves. On the other hand, gel theory suggests that plasticization is the result of interference between polymer interactions due to the presence of hydrogen, van der Waals, or ionic bonds. Meanwhile, the third of the aforementioned theories—free volume theory—assumes that the free volume between polymer chains increases due to the addition of a plasticizer, causing a lower glass transition temperature. An effective plasticizer should be hydrophilic and polar with low molecular weight so as to effectively penetrate the inter-chain polymer areas. In addition, it should have a boiling point higher than the process or treatment being carried out, so that it does not evaporate during it [184].

Blending TPS with polyesters or other plastics in the vast majority of cases improves mechanical properties and hydrophobicity, as well as resistance to biotic and abiotic factors. Due to the fact that we can isolate starch from many botanical sources, obtaining thermoplastic starch is a relatively inexpensive way of producing biodegradable and renewable products. Another advantage of TPS is that it can be obtained with the same techniques as commercial plastics (extrusion, injection, and vacuum molding), which can be an alternative to the use of resistant or poorly degradable plastics, especially in applications with a short life [4,47]. However, what is worth remembering, a definite disadvantage of plasticization is that the use of a hygroscopic plasticizer strengthens the water absorption of the final product. Therefore, due to the strongly hydrophilic nature, increased by the use of a plasticizer, TPS alone, similar to a starch, was not suitable for packaging material [183].

The joining of polymers to produce packaging material can take place not only by mixing but also by forming layers, i.e., layering. In recent decades, coextrusion has been widely used to combine two or more polymers into multilayer coatings. Multilayer products composed of TPS and polyesters can also be produced in the coating and press molding processes. Scientific and industrial research on thermoplastic starch mainly concerned mixing plasticized starch with hydrophobic biodegradable polyesters with much better mechanical properties, type PLA, PCL, poly (ethylene adipate) (PEA), poly (3-hydroxybutyrate-*co*-3-hydroxyoctanoate) (PHBO), poly (3-hydroxybutyrate-*co*-3-hydroxyvalerate) (PHBV), PHEE, and poly (succinate-*co*-adipate butylene) (PBSA) [182,195,196]. Wang et al. developed a method for obtaining biodegradable materials from starch-grafted polymers that show a wide application spectrum. LDPE and granular starch were mixed in the presence of an interphase compatibilizer (maleic anhydride) but without the use of glycerol as a plasticizer [197]. The resulting blends showed similar mechanical properties as the pure polymer, which resulted from the fact that the starch was chemically grafted on a physically interacting compatibilizer. The compatibility is carried out to improve interphase miscibility. Usually a compatibilizer is obtained by modifying one of the polymer components of the original blend. These modifications in starch-based mixtures are most often carried out by crosslinking starch/polyester with polyisocyanates or peroxides, functionalizing polyesters with anhydrides or synthesis of polyester phosphates, starch functionalization with urethane derivatives or polyglycidyl methacrylate, or the synthesis of starch-grafted copolymers. In cases where the compatibilizer is anhydrides and the mixture is a hydrophobic polymer and hydrophilic starch, their anhydride groups will react and covalently bind to the hydroxyl groups of the starch granules to form ester bonds [197]. The most effective interphase compatibility in a non-miscible two- or several-component system is obtained when the polymer component of the compatibilizer is identical to the polymer mixed with starch granules [198]. For example, the ideal compatibilizer for polypropylene will be polypropylene grafted with maleic anhydride (PP-g-MA). If you mix starch with a hydrophilic biodegradable polymer such as polyethylene oxide (PEG, polyethylene glycol), then the presence of a compatibilizer is no longer required for the covalent attachment of polyethylene to granular starch, as described by Loercks et al. [199]. In another experiment, Averous tested biodegradable mixtures based on wheat TPS with PCL and then extruded or injected the finished product to obtain a film [200]. In these studies, it was observed that the addition of even small amounts of PCL improved the processing properties of the material, i.e., reduced elasticity, technological shrinkage and sensitivity to moisture.

### 4.2. Non-Degradable or Weak Degradable Polymers for Starch-Based Packaging Materials

Already many years ago, a partially biodegradable blend of starch with LDPE was developed, which in time was particularly useful in the production of food packaging [201]. From this blend containing up to 50% of native or plasticized starch and unsaturated fatty acids as antioxidants, films have been successfully obtained by blowing extrusion. In other studies, injection molding and mechanical properties of potato starch or its phthalates (starch and phthalic acid esters) from LDPE were studied [202]. The esterification of starch made it possible to obtain mixtures exhibiting the characteristics of a thermoplastic material, with increased hydrophobicity, thermal stability, and after adding PE with better mechanical properties than in the case of non-esterified starch. Blends and composites of mechanically damaged starch granules (pulverized solid-state powder) with PE [203] or starch/PP [204] composites were also developed. In turn, Bhattacharya extruded, in a corotating twin-screw system, three-component blends with a majority (70%) of starch, 25% HDPE content, and a small addition of functionalized polymers like ethylene methacrylic acid copolymer (EMA) or ethylene vinyl acetate maleic anhydride copolymer (EVAMA) [205]. Maleic anhydride was used as a compatibilizer in the studies. Corn starch and LDPE [206] films as well as wheat starch and HDPE [207] were extruded and tested for strength. Other procedures for obtaining starch blends made of PE were developed by Ramkumar et al., where, as an additive facilitating interfacial interactions, they used in addition to those already known in the processing of poly (vinyl acetate) (EVA), EMA, and EVAMA, also cellulosic fibers [198]. One of the first, unfortunately unsuccessful, attempts to mix granular potato starch with aliphatic polyesters, type ammonium allylpolyethoxy sulfate (APES) and polyolefins of LDPE type was carried out by Park et al. [196]. Processing of blends based on wheat starch and PEAs in the presence of plasticizers such as water and glycerol was carried out by Averous et al. [208]. Chandra and Rustgi also used the anhydride of this acid in the preparation of LLDPE starch mixtures [209]. This time, maleic anhydride (MA) was grafted to LLDPE in the presence of an initiator, dicumyl peroxide (DCP). This treatment was necessary to ensure compatibility between synthetic polymers and starch, so that polar functional groups could interact with each other. This type of mixing allowed an increase in strength and Young’s modulus, and the percentage elongation decreased with an increase in the content of the polysaccharide component.

Matzinos et al. using conventional extrusion, injection molding, and film blowing techniques, also processed ternary blends of LDPE/TPS/PCL. The test results confirmed that the structure and properties of the mix depend not only on the composition but also on the processing method. In the films, the fine dispersion of the polycaprolactone phase in the polyethylene/starch matrix caused an increase in mechanical properties [210]. Using the coextrusion process, it was also possible to obtain multilayer films based on low-density polyethylene (LDPE) and potato and corn thermoplastic starch [211].

### 4.3. Biodegradable Polymers for Starch-Based Packaging Materials

Generally, to convert native starch or a derivative thereof into a thermoplastic form, it is sufficient to use a hydrophilic biodegradable polymer such as an aliphatic polyester, polyester amide or urethane, polyethylene oxide, or mixtures thereof.

Starch films with increased moisture resistance can also be obtained by preparing multilayer sandwich-type structures with a central thermoplastic starch layer and hydrophobic components on the outer layers [47]. However, the most common method for obtaining biodegradable multilayer starch-based films is coextrusion. Despite many disadvantages such as interfacial instability and uneven layer distribution, it is a method that allows for coextrusion of starch with PLA, PCL, PBSA, PHBV, PBAT, and PEA polymers in one step and a continuous manner. Martin et al. coextruded and examined the properties of biodegradable multilayer films based on plasticized wheat starch [200].

In order to maintain biodegradability of mixtures with thermoplastic starch, a number of types of blends with other natural polymers such as pectins or more often biodegradable polyesters such as PHBV, PCL, PLA, PEAs, or PBSA were carried out. The obtained blends were characterized by improved mechanical properties, increased hydrophobicity, and water-vapor impermeability. Their disadvantage was still poor compatibility and poor surface properties [212]. Hence, subsequent proposals of research groups focused on the use of additives in the form of compatibilizers or preliminary derivatization of starch [213]. A good solution enabling the improvement of processing properties turned out to be the use of starch-based composites and one of the few H_2_O soluble polymers for production, which is poly (vinyl alcohol). The materials thus obtained were characterized by high flexibility and strength, which was the result of relatively good mechanical compatibility with a rather low degree of miscibility and formation of so-called continuous polymer phase [214]. The mechanical properties of the mixtures produced by various research groups depended not only on their composition but also on the processing method; while in the vast majority of examples presented, the result of increasing the content of the polysaccharide component was the deterioration of mechanical parameters. The general trend is that the better the dispersion of starch in a thermoplastic matrix, the better the mechanical properties of the final product. In fact, the addition of starch generally increases stiffness. This corresponds to lower tensile strength and reduced elongation at break.

Attempts have also been made to produce packaging materials based on hybrid starches, i.e., mixed starches from various botanical sources. Therefore, the focus was mainly on starches with an increased content of amylose, i.e., corn and rice starch, to create a hybrid polymer material, with increased mechanical resistance and improved processing properties [215].

Extrusion of nanocomposite films is another approach in the manufacture of starch packaging materials. Nanocomposites refer to materials with a heterogeneous structure, whose individual components do not dissolve in relation to each other and show different properties. Most often, nanocomposites in starch-based packaging are in the form of nanofiller. They are introduced into the system not only to improve mechanical strength and increase the barrier to gases and steam but also to reduce production costs by reducing the consumption of the more expensive main component. Nanocomposites based on starch and clay are already known, serving as a starting material for the development of biodegradable packaging films [216]. Nanoclays filling composite layers improved transport properties and mechanical strength. In subsequent studies, nanosilica and rice husk ash served as a filler in a nanocomposite based on corn starch [217]. Zeolites are other compounds that can successfully serve as fillers in starch films. These microporous aluminosilicate minerals affect the properties of the entire composite by reducing its water solubility and increasing gas barrier and increasing the modulus of elasticity. Addition of thickener with silicate structure Laponite^®^, in turn, improves the mechanical and functional properties of packaging based on starch and PVOH, which due to their high compatibility and biodegradability can compete with packaging obtained from petroleum polymers [218]. Films based on a small amount of corn starch (up to 20%), citric acid, and carboxymethyl cellulose were also obtained by using the casting method. In such composites, interactions between the carboxylic acid groups and the hydroxyl groups of starch lead to a decrease in the availability of the latter and thus improve moisture resistance. In turn, cellulose fibers, used as a biodegradable filler in starch composite, additionally improve its mechanical properties, such as tear strength. Chemical similarities in the structure of starch and cellulose ensure good interaction [219].

Another example was starch grafted on vinyl emulsion, which was biodegradable and can be used as a sustainable packaging material [220]. Biodegradable packaging material based on starch was also obtained by using multicomponent mixtures. This blend consisting of corn starch, PCL and PHB has been prepared using a twin-screw extruder. Although PCL has good processing properties, it is expensive, and PHB, although it has a high tensile strength, is relatively fragile and poorly processable. Due to the poor compatibility of ingredients and low processability in most of them, it was necessary to use additional compounds such as water and glycerol plasticizers for starch and triacetin for PHB, as well as agents that facilitate the mixing of a mixture of stearic and palmitic acid mixtures called stearin III. As a result, cheaper and biodegradable packaging material with sufficiently good mechanical properties was obtained [221].

In turn, Fang et al. focused on the use of modified starch film (acylated potato starch) as a laminate component in combination with other polymers such as PLA in one of their papers [222]. Based on the esterification of starch with oleic and linoleic acids, they concluded that the higher the number of double (unsaturated) bonds in esters, the better their thermoplastic properties. Research on the processing capacity and subsequent strength of other composites based on starch, this time rice, PLA, and epoxidized natural rubber, was also carried out [223]. In addition, as part of assessing the properties of the resulting composites, it determined water absorption and susceptibility to enzymatic degradation.

The applicability of starch was significantly improved by the use of a non-toxic, biocompatible, and biodegradable other polysaccharide, namely chitosan. The influence of chitosan concentration and electron beam (EB) radiation on the mechanical, processing and microbiological properties of films prepared on the basis of starch was investigated. It turned out that chitosan starch mixtures can be not only good and biodegradable packaging material but also antibacterial by Zhai et al. [224]. In other studies, biodegradable films based on mixtures of rice starch and chitosan were successfully developed by applying the casting method. The films with the addition of chitosan showed an increase in tensile strength and a decrease in elongation at break, solubility, and water-vapor permeability [225]. Bourtoom also checked the effect of various plasticizers (glycerol, sorbitol, and polyethylene glycol) on the mechanical properties, solubility, and barrier properties of the film based on rice starch and chitosan. The increase in the concentration of the plasticizer was synonymous with an increase in solubility and permeability but also with a decrease in tensile strength [226]. In turn, Li et al. compared the properties of films based on unmodified and crosslinked chitosan/starch composites. The crosslinking agent was glutaraldehyde, and the films were prepared by solvent evaporation. Crosslinking deteriorated the compatibility of the composites and thus the mechanical properties with increased water barrier properties [227]. Mendes et al. obtained polymer blends based on thermoplastic corn starch and thermoplastic chitosan (TPC) by extrusion process. The use in TPC blends allowed for the production of a film characterized by higher thermal stability but with reduced tensile strength [228]. The effect of chitosan concentration on the mechanical and barrier properties was also investigated in the film produced by the casting method from corn starch. The addition of chitosan, as was the case in previous reports, improved the mechanical strength and reduced the water-vapor permeability of the final material [229]. Multicomponent composites based on TPS were also obtained with the addition of such unusual ingredients as chicken feathers (as a source of keratin). A styrene-butadiene elastomer/thermoplastic starch (TPS)/ethylene vinyl alcohol copolymer, and chicken feathers (EB/TPS/EVOH/CF) composites were prepared by melt blending. The presence of styrene in the composite contributed to the increase in plasticity, and the feather keratin improved the thermal stability of the final product [230].

In addition to polysaccharides such as cellulose or chitosan, proteins have also been used to improve the properties of starch. Corradini at al. used zein in thermoplastic starch compositions. Materials based on blends of glycerol plasticized starch and corn protein were prepared by melting processing at 160 °C and then by compression molding. Despite of blend components incompatibility, the use of zein in TPS composites improved the processing properties such lower water sensitivity and lower viscosity [231].

Year by year, more and more attention has been paid to producing only biodegradable blends of biopolymers derived from renewable sources. The results have not always been promising. TPS and PLA blends with glycerol by injection molding and also by compression molding for comparison were prepared. The structure analysis confirmed the weak interaction of the components and heterogenous final material. The higher concentration of TPS caused a decrease in mechanical strength and hydrophobicity [232]. Other research group prepared of binary and ternary blends like PCL/PLA and PCL/PLA/TPS, respectively. The addition of TPS decreased the thermal stability of the PCL/TPS and PLA/TPS blends. On the other hand, the increase in TPS content in PLA/PCL blends (in-ternary system) deteriorated the mechanical properties, which was explained by low compatibility [233]. Some studies were performed to evaluate the performance of blends of PBS with two different types of TPS, EGTPS (plasticized with glycol), and GTPS (plasticized with glycerol), in different compositions for potential packaging applications [234]. The glycerol was better plasticizer. However, it was confirmed, that the PBS/TPS polymers were immiscible and had poor compatibility, and the greater addition of TPS deteriorated the system mechanical properties. Ternary starch blends with PHBV and polyethylene oxide (PEO) as compatibilizer or PEO-coated starch in PHBV did not bring improvement or hope for obtaining more durable materials. Even mixing PHB with three different starch derivatives: native starch, starch adipate, and starch-grafted urethane resulted in still brittle materials. Only after plasticizing starch with a high glycerol content and mixing with PHBV and PBAT, a material with very good mechanical properties was obtained, ideal for flexible packaging. It was found that the starch phase is miscible with the PHBV and PBAT phases, thus acting as a compatibilizer in the system [235]. In addition to developing increasingly compatible blends of starch or TPS with other biodegradable polymers, effective modification methods are still being sought. Recently, a lot of reports have appeared on new methods of obtaining packaging materials by various methods, not only casting. Starch-cellulose films have been successfully produced by tape casting using three different drying procedures: conduction, infrared conduction, and infrared drying [236]. Multilayer films based on starch, for example, are widely researched and produced by compression molding. In one study, the monolayer was cassava starch combined with polyester blends containing carvacrol, essential oil, chitosan, or PLA [237]. In other studies, Zarski et al. extruded a film based on plasticized potato starch esters with better mechanical properties compared to non-esterified starch [238]. In turn, with the use of a twin-screw extruder, a sustainable, intelligent packaging based on cassava and anthocyanin starch [239], as well as nanocomposites with thermoplastic corn starch (TPS) and cellulose nanofibers (CNF) as an enhancer [240] have already been developed. There are also known studies in which also developed an antibacterial film consisting of TPS, covered with a gelatin coating and polybutylene adipate terephthalate (PBAT) using this method [241].

Currently, crosslinking reactions are most often used in the preparation of starch materials by reactive extrusion. In some studies, on the other hand, films based on crosslinked maize starch were extruded in a reaction catalyzed by chromium octanoate with cellulose acetate as filler and glycerol as plasticizer, respectively. In both cases, crosslinking during reactive extrusion resulted in a film with lower water absorption and better mechanical properties [242]. Another example of this is the crosslinking of cassava starch with citric acid in the presence of sodium hypophosphite catalyst and glycerol as plasticizer [243].

Acetylated cassava starch and pea protein isolate were used in the production of blown film. The applied isolate increased the thermal stability, tensile strength, hydrophobicity, and barrier to oxygen and water vapor [244]. Extrusion blow molding has also turned out to be a good method to obtain nanocomposite starch films. Apart from glycerol, the components of the film were simple and complex sugars, which mainly contributed to increasing its flexibility [245].

## 5. Conclusions: Challenges and Future Perspectives

The times when starch was associated only with food are over. This is due to the wide availability and renewal of this biopolymer. Its low production price, non-toxicity, and biodegradability are features that have attracted the attention of non-food industry branches, mainly packaging producers, for many years. Although native or plasticized starch, mainly due to its hydrophilicity and poor mechanical properties, is not an ideal packaging material, various research centers continue to intensify activities toward its processability and functionality. A wide range of physical, chemical, and other modifications makes it possible to increasingly give it properties similar or even competitive to petroleum polymers.

Starch and materials based on starch or its derivatives in a system with other natural or synthetic polymers, but only degradable, may constitute the future for sustainable packaging. The commercialization and increase in demand for this type of materials, however, requires certain measures, such as reduced production costs, mainly in biodegradable low-starch blends with polyesters, increased miscibility, as well as eliminated deterioration of mechanical and performance properties in high-starch blends, also those treated with a compatibilizer or based on hydrophobic derivatives. The esterification of starch with fatty acids to increase hydrophobicity and improve thermoplasticity seems to be a significant modification that can increase the usefulness of this polysaccharide in packaging production. It can be found more and more reports on the production of starch-based materials by methods less typically laboratory and more industrial. This is a good prognosis, because not every material obtained, e.g., by casting method, can work under the conditions necessary for effective industrial production.

Methods based not only on compression or injection molding and simple extrusion, but such as co-, blown-, or reactive-extrusion are attracting more and more attention. What is very important is that functionalization of starch-based materials toward packaging is developing day by day, not giving way to other more popular polymers.

Even if native or plasticized starch itself does not show such processing and mechanical properties to compete with widely distributed synthetic polymers, it is worth emphasizing that their modification, primarily through hydrophobization, will allow one not only to obtain thermoplastic material but more importantly material with increased dispersibility in other hydrophobic thermoplastic materials. As a result, it is possible to design materials with increased biodegradability while maintaining sufficiently good processing and mechanical properties and use pro-ecological modification conditions, ensuring the materials are obtained in accordance with the green chemistry and principles of sustainable development.

## Figures and Tables

**Table 1:** Selected chemical modifications and changes in starch properties.

Type of Chemical Modification	Properties of Modified Starch	Ref.
Cross-linking	- higher thermal stability and shear - lower swelling force - better stability during freezing and thawing	[93,94,95]
Grafting polymerization	- stronger absorption of H_2_O - better thermal stability - increase in compatibility - increase in biodegradability	[5,96,97]
Oxidation	- increase in solubility - higher viscosity and gelatinization temperature - stronger absorption of H_2_O	[5,98,99,100]
Etherification	- better thermal stability - increase in solubility - better durability - increase in permeability	[101,102,103,104,105,106]
Esterification	- increase in compatibility - increase in thermoplasticity - better resistance to moisture - decrease or increase in thermal stability - worse biodegradability	[7,8]

**Table 2:** Modification of starch under microwave radiation.

Starch	Reagent	Solvent	Catalyst	Power [W]	Product	Ref.
Cassava starch	Dimethyl carbonate	Sodium chloride	Na_2_HPO_4_	1300	Methylated starch	[133]
Cassava starch	Dimethyl carbonate	Dimethyl carbonate	Na_2_HPO_4_ NH_2_CONH_2_ NH_2_CSNH_2_	800	Methylated starch	[134]
Corn starch	Acetic acid anhydride	Acetic acid anhydride	I_2_	300	Starch acetate	[130]
Corn starch	Acetic acid anhydride	Acetic acid anhydride	I_2_	300	Starch acetate	[131]
Corn starch	Vinyl acetate	H_2_O	K_2_CO_3_	n/d	Starch acetate	[135]
Maize starch	Oleic acid	H_2_O	*Staphylococcus aureus* lipase	700	Starch oleate	[136]
Cassava starch	Octenyl succinic anhydride	H_2_O	NaOH	50	Starch octenyl succinate	[137]
Potato starch	Oleic acid	-	*Candida* *antarctica* lipase	105–315	Starch oleate	[132]

**Table 3:** Esterification of starch in ionic liquids (ILs).

Starch	Ionic Liquid	Catalyst	Product	Ref.
Corn starch	[BMIM]Cl	Pyridine	Starch acetate or succinate	[147]
Corn starch	[BMIM]Cl	-	Starch acetate	[148]
Corn starch	Imidazolium based ILs	ILs	Starch mixed esters	[149]
Corn starch	[BMIM]Cl	[BMIM]Cl	Starch acetate or propionate	[150]
Corn maltodextrin	[BMIM]X; X-halogen	-	Maltodextrin acetate	[151]
Corn starch	IL, DMF or DMSO	-	Starch laurate, palmitate or stearate	[152]
Corn starch	[BMIM]Cl	Pyridine	Starch laurate or stearate	[153]
Cassava starch	[BMIM]Cl	Pyridine	Starch vernolate	[154]
High-amylose maize starch	Mixture of [BMIM]BF_4_ [BMIM]OAc	*C. rugosa* lipase	Starch palmitate	[155]
High-amylose maize starch	[BMIM]BF_4_	*C. rugosa* lipase	Starch laurate	[156]
Cassava starch	Mixture of [BMIM]PF_6_ DMSO	Novozyme 435 lipase	Starch vernolate	[157]
Waxy maize starch	[C_8_MIM]NO_3_	Novozyme 435 lipase	Starch octenyl succinate	[158]
Corn starch	C_16_ MIMBr, C_16_-3-C_16_ IMBr_2_, C_16_-12-C_16_ IMBr_2_	*R. oryzae* lipase	Starch oleate	[159]
Potato starch	[BMIM]Cl	*T. lanuginosus* lipase	Starch oleate	[160]
Potato starch	[BMIM]Cl P80 surfactant	*T. lanuginosus* lipase	Starch esters	[161]

**Table 4:** Esterification or transesterification of starch in supercritical CO_2_.

Starch	Reagent	Catalyst	Product	Ref.
Potato starch	Acetate anhydride	Sodium acetate	Starch acetate	[162]
Potato starch	Acetate anhydride	K_2_CO_3_	Starch acetate	[163]
Potato starch	Acetate anhydride	Sodium acetate	Starch acetate	[164]
Potato starch	Anhydride or methyl/vinyl esters	K_2_CO_3_	Starch esters	[165]
Sago starch	Methyl esters	K_2_CO_3_	Starch esters	[166]

**Table 5:** Modification of starch by mechanochemistry method.

Starch	Reagent	Solvent	Catalyst	Product	Ref.
Waxy rice starch	Octenyl succinate anhydride	H_2_O	NaOH	Starch octenyl succinate	[169]
Cassava starch	Octenyl succinate anhydride	-	Na_2_CO_3_	Starch octenyl succinate	[170]
Cassava starch	Lauric acid	-	K_2_CO_3_	Starch laurate	[171]
Waxy rice starch	Octenyl succinate anhydride	H_2_O	NaOH	Starch octenyl succinate	[172]

**Table 6:** Advantages and disadvantages of the most commonly used media and techniques in starch modification.

Methods of Starch Modification	Medium or Technique of Modification			Ref.
	Name	Advantages	Disadvantages	
Physical, chemical, and biochemical	organic/inorganic solvents	+ low production cost + usually easy recycling + wide temperature range of application	- toxicity - volatility - usually flammability - high temperatures and long times of reaction	[8,102]
Physical, chemical, and biochemical	ionic liquids	+ non-volatility + non-flammability + multifunctionality + designer solvents	- high cost of production - difficult purification and recycling - usually toxic	[144,145,146]
Physical and chemical	supercritical CO_2_	+ easy recycling + non-toxicity + non-flammability + non-volatility + multifunctionality	- high cost of production - purification problems	[159,160,161,162,163]
Physical, chemical, and biochemical	microwave radiation	+ reduction of reaction time	- high cost of apparatus	[8,132]
Physical and chemical	ultrasonication	+ reduction of reaction time	- high cost of apparatus	[164,165]
Physical and chemical	mechanochemistry	+ reduction of reaction time + solvent-free reactions	- high cost of apparatus	[166,167,168,169]

**Table 7:** The examples of starch/thermoplastic starch blends with reinforcements or fillers.

Composition of Matrix	Reinforcement or Filler	Ref.
TPS/PVA	Softwood fiber	[185]
Potato starch/Cellulose derivatives/Synthetic polymers	Flax, jute, ramie, oil palm fiber	[186]
Corn starch/PCL	Flax and ramie	[186]
Wheat starch/PCL	Flax and ramie	[186]
Wheat starch/Glycerol/Sorbitol/TPS	Flax and ramie	[186]
TPS/ Thermoplastic PU	Flax	[187]
TPS/PCL	Flax	[187]
Maize starch/TPS	Flax fiber	[187]
TPS/PCL/EVOH	Non-woven of flax, hemp, ramie fibers	[188]
Starch/Glycerol/Formamide/Urea	Micro winceyette fiber	[189]
Wheat starch/Glycerol/TPS	Leafwood fibers	[190]
Starch/EVOH	Hydroxylapatite-reinforced	[191]
Maize starch/Glycerol	Kaolin	[192]
TPS	Montmorylonit, Cloisite 30B	[193]
TPS/PLA	Sugar palm nanocellulose fibers	[194]

## Data Availability

Not applicable.
